# Non-canonical Notch signaling represents an ancestral mechanism to regulate neural differentiation

**DOI:** 10.1186/2041-9139-5-30

**Published:** 2014-09-19

**Authors:** Michael J Layden, Mark Q Martindale

**Affiliations:** 1 Department of Biological Sciences, Lehigh University, Bethlehem, PA 18015, USA; 2 Whitney Laboratory for Marine Bioscience, University of Florida, St Augustine, FL 32080, USA

**Keywords:** Notch, *Nvnotch*, *Nematostella vectensis*, Cellular differentiation, Evolution

## Abstract

**Background:**

Cellular differentiation is a critical process during development of multicellular animals that must be tightly controlled in order to avoid precocious differentiation or failed generation of differentiated cell types. Research in flies, vertebrates, and nematodes has led to the identification of a conserved role for Notch signaling as a mechanism to regulate cellular differentiation regardless of tissue/cell type. Notch signaling can occur through a canonical pathway that results in the activation of *hes* gene expression by a complex consisting of the Notch intracellular domain, SuH, and the Mastermind co-activator. Alternatively, Notch signaling can occur via a non-canonical mechanism that does not require SuH or activation of *hes* gene expression. Regardless of which mechanism is being used, high Notch activity generally inhibits further differentiation, while low Notch activity promotes differentiation. Flies, vertebrates, and nematodes are all bilaterians, and it is therefore unclear if Notch regulation of differentiation is a bilaterian innovation, or if it represents a more ancient mechanism in animals.

**Results:**

To reconstruct the ancestral function of Notch signaling we investigate Notch function in a non-bilaterian animal, the sea anemone *Nematostella vectensis* (Cnidaria)*.* Morpholino or pharmacological knockdown of *Nvnotch* causes increased expression of the neural differentiation gene *NvashA*. Conversely, overactivation of Notch activity resulting from overexpression of the *Nvnotch* intracellular domain or by overexpression of the Notch ligand *Nvdelta* suppresses *NvashA.* We also knocked down or overactivated components of the canonical Notch signaling pathway. We disrupted *NvsuH* with morpholino or by overexpressing a dominant negative *NvsuH* construct. We saw no change in expression levels for *Nvhes* genes or *NvashA.* Overexpression of *Nvhes* genes did not alter *NvashA* expression levels. Lastly, we tested additional markers associated with neuronal differentiation and observed that non-canonical Notch signaling broadly suppresses neural differentiation in *Nematostella*.

**Conclusions:**

We conclude that one ancestral role for Notch in metazoans was to regulate neural differentiation*.* Remarkably, we found no evidence for a functional canonical Notch pathway during *Nematostella* embryogenesis, suggesting that the non-canonical *hes*-independent Notch signaling mechanism may represent an ancestral Notch signaling pathway.

## Background

Metazoan development requires a mechanism to control the balance between pools of cells that are able to differentiate into distinct specialized cell types and cells that remain undifferentiated to contribute to growth or differentiate at a later time. Identifying the mechanisms that regulate this balance provides insights into the evolution of animal developmental programs and clues as to the putative molecular changes that underscored the emergence of metazoans from single-celled ancestors. Functional studies have identified the Notch signaling pathway (described below) as a conserved regulator of cellular differentiation, but this is only known from bilaterian animals. There are at least four metazoan lineages that diverged prior to the emergence of bilaterians. They are the ctenophores, poriferans, placozoans, and cnidarians, with cnidarians being the most closely related to bilaterians [1-4].

Notch signaling is implicated as a regulator of cellular differentiation in multiple bilaterian tissue types including neural, blood, epidermal, endothelial, muscle, and bone [5-10]. A well known and studied example of Notch regulation of differentiation is in bilaterian neurogenesis. During the formation of the *Drosophila* ventral nerve cord, cells with high Notch activity suppress the formation of a neuroblast progenitor cell in favor of maintaining undifferentiated neural ectoderm fate [11,12]. Similarly, in vertebrate neurogenesis, high Notch activity in neural stem cells acts to maintain a neural stem cell fate identity, while low notch activity in daughter cells promotes neuronal differentiation [8,13]. In both vertebrate and invertebrate neurogenesis, Notch inhibits neurogenesis by repressing the expression of proneural gene transcription factors [11,13-15]. Proneural gene transcription factors are basic helix-loop-helix transcription factors that belong to either the *achaete-scute* or *atonal* gene families [15].

There are two mechanisms by which Notch can regulate differentiation. They are the canonical [16] and non-canonical pathway [16,17]. The core minimal components shared by both pathways are the *notch* receptor, *delta* ligand, and the γ-secretase and ADAM protease cleavage complexes [9,16]. Additional core components required specifically for canonical Notch signaling are *hes*, *suH*, *smrt,* and *mastermind*[9,18,19]. Both canonical and non-canonical pathways are typically initiated by the binding of Delta to the Notch receptor, which induces cleavage and release of the Notch intracellular domain by Adam protease and γ-secretase cleavage events. In the canonical pathway, the Notch intracellular domain interacts with SuH and displaces the SMRT co-repressor normally bound to SuH and recruits the Mastermind transcriptional co-activator. This complex then induces expression of the *hes* genes, which regulate expression of Notch targets, such as the proneural genes [9,16,18]. Non-canonical Notch signaling bypasses interactions with SuH and activation of *hes* gene expression, to regulate target gene expression via alternative mechanisms [16,20].

Genomic analyses of core conserved Notch components suggest that the core Notch pathway evolved specifically in the metazoan lineage. *notch, delta,* and *hes* homologs do not exist outside of the metazoans [19,21], and all five major animal clades possess a Notch homolog. The ctenophores are the only non-bilaterians lacking a definitive Delta ligand, although they possess many Delta-like proteins that could potentially activate Notch ligands [1,19], and, recently, Delta-like genes have been identified to activate Notch in bilaterians [22]. Of the remaining core conserved genes, the members of the γ-secretase complex and ADAM proteases all predate the metazoan divergence [19]. Key regulatory components of the canonical Notch pathway were not present until the emergence of the cnidarian-bilaterian common ancestor. The *suH* gene evolved prior to the earliest metazoans, but the *mastermind* co-activator evolved in the cnidarian-bilaterian ancestor [19], and the SMRT co-repressor is not present outside of the bilaterian lineage [1,3,4,19,23,24]. Thus, although Notch signaling evolved early in the metazoan lineage, it is unclear if the canonical or non-canonical pathway represents the ancestral Notch signaling mechanism.

One way to determine the evolution of a particular signaling pathway is to determine how it functions in phylogenetically informative extant animals that allow reconstruction of the ancestral role(s) of the pathway at deep evolutionary nodes within the animal phylogeny. However, gene-specific functional studies addressing Notch signaling in non-bilaterian metazoans is currently lacking. Characterization of the expression patterns of Notch signaling components and pharmacological disruption of γ-secretase implicate Notch as a regulator of differentiation in the non-bilaterians [25-28]. First, in the poriferan *Amphimedon queenslandica*, *Amqdelta* homologs are expressed in differentiating cell types throughout development [25]. In the cnidarians, treatment with DAPT, which inhibits γ-secretase cleavage of the Notch intracellular domain [26,28,29], increases expression of differentiated cell markers (particularly neuronal markers) [26]. One of these markers is *NvashA*, which is an *achaete-scute* gene family homolog known to regulate embryonic neurogenesis in *Nematostella*[30]. Marlow and coworkers [26] also investigated the role of *NvsuH* on development of the cnidocytes, which are the stinging cells in *Nematostella*, using a splice blocking morpholino (MO) against the *NvsuH* gene and a dominant negative construct. They found that mature cnidocytes were lacking in *Nematostella* planula when *NvsuH* function was reduced and that this phenotype was similar to the reduction in cnidocytes resulting from treating animals with DAPT [26]. However, in this study the authors did not compare other phenotypes resulting from DAPT treatment with a disruption of *NvsuH*. DAPT treatment has been found to inhibit maturation, but not specification, of cnidocytes in polyps of the hydrozoan cnidarian *Hydra*[28]. Taken together, the previous studies in non-bilaterians suggest that Notch signaling played a role in regulating the process of neuronal cell differentiation in the cnidarian-bilaterian ancestor, but the lack of detailed gene-specific studies does not clarify if the canonical or non-canonical Notch signaling pathway represents the ancestral mechanism of Notch signaling.

Here we take advantage of the ability to conduct functional genetic experiments in the cnidarian sea anemone *Nematostella vectensis* to characterize the role of Notch signaling during embryonic development. We show that Notch activity in *Nematostella* suppresses expression of *NvashA*-dependent neural differentiation markers [30], and that the suppression of *NvashA*-dependent neural markers occurs via specific inhibition of *NvashA* expression by *Nvnotch*. We also show that Notch activity broadly inhibits expression of neuronal differentiation markers for other neural cell types in the *Nematostella* embryo. Although some components of canonical Notch signaling are present in the *Nematostella* genome, our experiments indicate that inhibition of differentiated cell markers occurs via the non-canonical (*Nvhes*-independent) mechanism during embryonic development.

## Methods

### Genes used in this study

The genes used in this study were previously published [26,30,31].

### Embryo manipulations and *in situ* analysis

All embryos were grown to either early gastrula stages, by raising animals for 24 hours post-fertilization (hpf) at 17°C, or to late gastrula stages, by raising animals for 24 hpf at 25°C. All fixation, *in situ* probe synthesis, and *in situ* hybridizations were carried out as previously described [30,32,33]. Images were obtained on a Zeiss Imager M2 in conjunction with the Axiocam HRc and ZenPro software (Carl Zeiss LLC, Thornwood, NY, USA). For gastrula stage analysis, 10 μM DAPT treatment was begun 3 hpf as previously described [26]. For larval stage analysis of DAPT-treated animals, animals were allowed to grow to desired stage (either 24 or 48 hpf at 25°C) and then, animals were incubated in 10 μM DAPT for 24 hours.

### mRNA injections

The *Nvnicd* fusion construct was generated by PCR amplifying the intracellular domain of *Nvnotch* using (Forward 5′ CACCATGGTTGTTGTGCTCGCAGGCGGTAAG 3′ and Reverse 5′ GTCTGATAATAACTCCACTATGTC 3′) PCR primers. The PCR product was then cloned *Nvnicd* in frame and 5′ to the *venus* coding sequence using the Gateway cloning vector system (Invitrogen, Carlsbad, CA, USA). Full length *Nvdelta* was amplified using the (Forward 5′CACCATGCAGCTACTACCACTCCAGCCATCAC 3′ and Reverse 5′ ATATTTCCACTTCCACTTCTTGCCAG 3′) primers and cloned in frame 5′ to the *venus* coding sequence. *Nvhes2* and *Nvhes3* constructs were cloned using (Forward 5′ CACCATGGAAAAAATGCGGAGGGCGAG 3′ and Reverse 5′ TCAAATTGTCCTCCCCATTCAC 3′) and (Forward 5′ CACCGCCGTTGACTGCATCGATAGC 3′ and Reverse 5′ TCACCATGGGCGCCACAGTG) PCR primers, respectively. They were both cloned in frame to the 3′ end of the *venus* coding sequence. Injection concentration of the *Nvnicd:venus* was 300 ng/μl. Injection concentration of *Nvdelta:venus* was 900 ng/μl. Injection concentrations of *venus:hes2* and *venus:hes3* were 300 ng/μl and 150 ng/μl, respectively. We injected the previously published *NvashA:venus* and *NvsuHDN:venus* mRNA as described previously (Layden and colleagues [30] and Marlow and colleagues [26]). mRNA was prepared and injected as previous described [30,34]. Animals were sorted prior to analysis to identify embryos expressing the Venus reporter protein and to eliminate the non-expressing animals.

### Morpholino injections

Fluorescein labeled *NvashA* translation-blocking MO was injected as published [30]. *NvSuH* splice-blocking MO was injected, and splice blocking was observed as previously described [26]. An *Nvnotch* splice-blocking MO (5′ GTCCTTTGATTTCGTACCTCATGGA 3′) (GeneTools Inc., Philomath, OR, USA) that results in a truncation of the *Nvnotch* intracellular domain and *Nvdelta* splice-blocking MO (5′ GCGACCTGACAAGAACAGTGAAGTC 3′) (GeneTools Inc.) that removes the exon containing the MNNL domain were designed and injected at 1 mM and 600 nM, respectively. Splice-blocking efficiency was estimated using PCR and DNA electrophoresis to observe shifts in the size of the wild-type or morphant mRNA. A control MO (5′ AGAGGAAGAATAACATACCCTGTCC 3′) was also injected at a concentration of 1 mM and gene expression was compared to uninjected control animals. Animals were sorted after injection to eliminate the uninjected animals as indicated by the lack of fluorescence.

### Quantification of cell number

To count the number of *NvashA*-expressing cells we mounted animals with the aboral end up, visualized using the 10× objective on the Zeiss Imager M2 (Carl Zeiss LLC). We normalized the focal plane by focusing on the most superficial level of the aboral ectoderm and then counted the total number of visible cells.

### Quantitative PCR analysis

RNA isolation and quantitative (q)PCR analyses were conducted as previously described [30]. *Nvactin*, *Nvef1B,* and *Nvatpsynthase* house-keeping genes were used to normalize fold change in qPCR experiments. All qPCR primers used have been previously described [26,30,35]. Each qPCR analysis was repeated in triplicate pools of embryos injected in independent sessions. Based on previous studies, we consider a fold change greater than 1.5 meaningful. We often fail to detect changes in expression via alternative approaches for fold-changes less than 1.5.

## Results

### *Nvnotch* and *Nvdelta* spatiotemporal expression is consistent with that of a regulator of cellular differentiation

**Figure 1:**
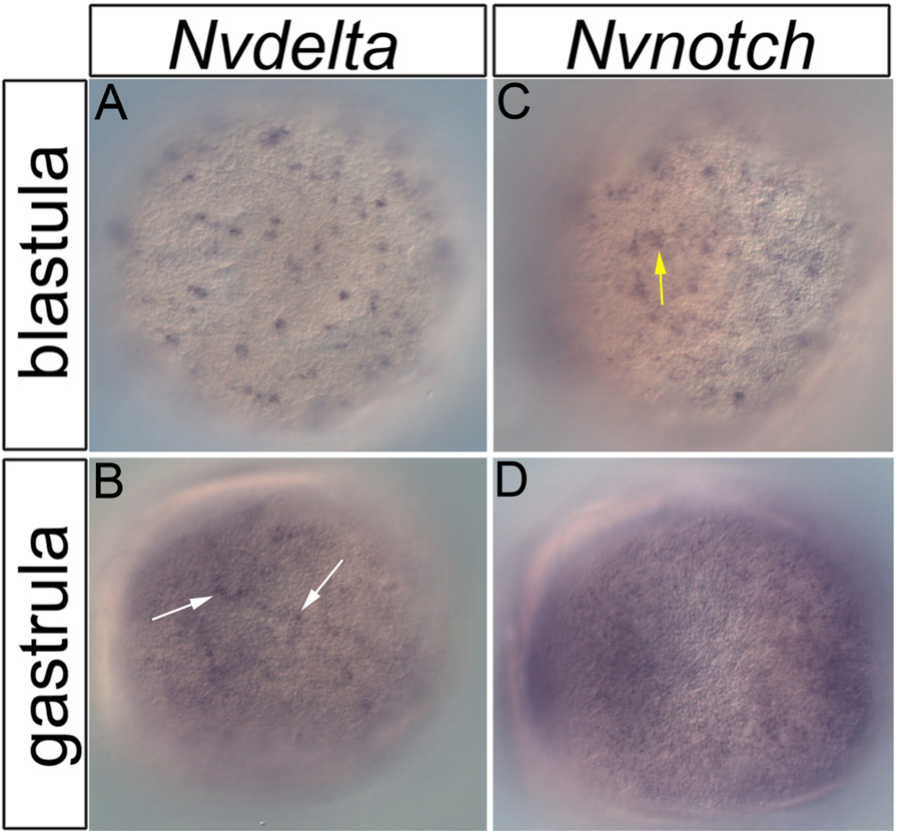
***Nvnotch *and *Nvdelta *embryonic expression.** Expression of *Nvdelta***(A,B)** and *Nvnotch***(C,D)** is shown at early gastrula **(A,C)** and late gastrula **(B,D)** stages. *Nvdelta* is expressed in a “salt and pepper” expression pattern at early gastrula **(A)**, and ubiquitously expressed at late gastrula **(B)**, though there are cells enriched for *Nvdelta* in the late gastrula (**B**, arrows). Clusters of cells distributed in a “salt and pepper” pattern express *Nvnotch* in the early gastrula stages **(C)**. By late gastrula, *Nvnotch* appears to have low-level ubiquitous expression **(D)**. Images are lateral views taken from a superficial focal plane; oral is to the left.

**Figure 2:**
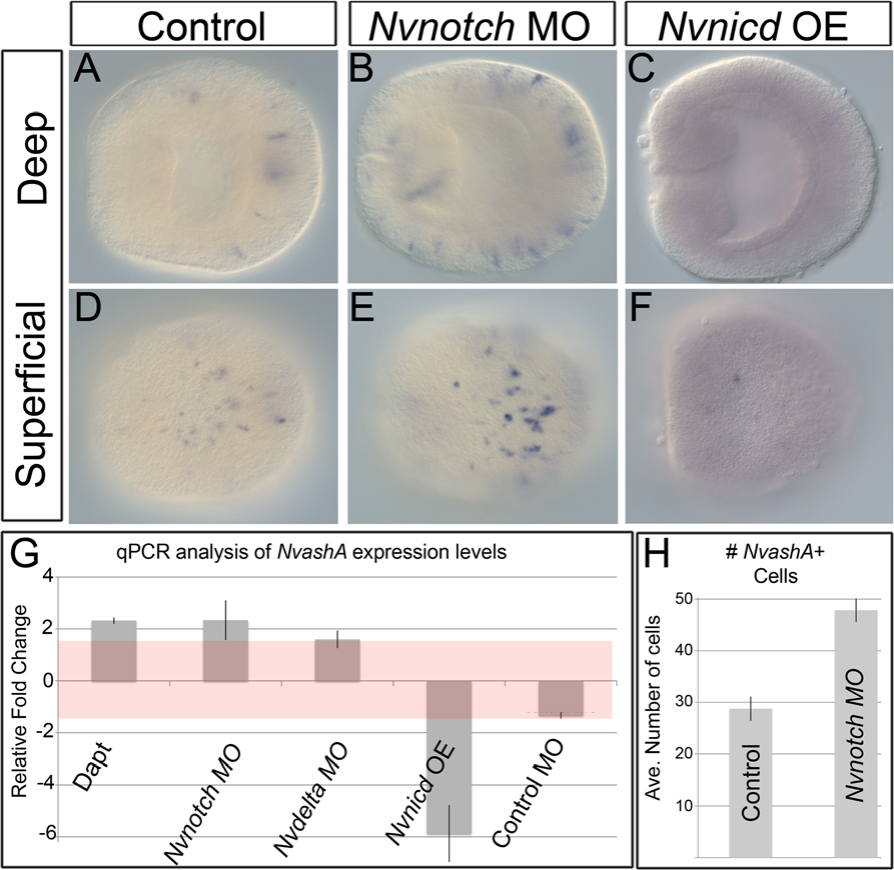
**Activation of *Nvnotch *suppresses *NvashA *expression.** Images of animals stained for *NvashA* by *in situ* hybridization are shown **(A-F)**. All images are lateral views with oral to the left. The relative focal plane is indicated to the left of each row of images. Animals with control wild-type Notch activity **(A,D),** with Notch activity reduced by injection of a *Nvnotch* morpholino (MO) **(B,E)**, and with Notch activity overactivated by overexpression (OE) of the *Nvnotch* intracellular domain (Nvnicd) **(C,F)** are shown. **(G)** Quantitative (q)PCR analysis of the relative expression of *NvashA* is compared in animals with reduced Notch activity (DAPT, *Nvnotch* MO, *Nvdelta* MO) and increased Notch activity (*Nvnicd* OE), and to animals injected with a control MO. The red rectangle indicates a relative fold change of −1.5 to 1.5, which we consider to correspond with no change in expression level. **(H)** Quantification of the average number of *NvashA*-positive cells counted in the aboral domain (see Methods). N ≥20 animals counted for each treatment.

Previous studies showed that *Nvnotch* and *Nvdelta* are expressed in tissues known to be undergoing differentiation in late gastrula through juvenile polyp developmental stages [26]. However, multiple studies suggest differentiation in *Nematostella* is first observed in the early gastrula when expression of neural genes *NvashA*[30] and *Nvelav*[31,36] are detected. We tested for both *Nvnotch* and *Nvdelta* expression by mRNA *in situ* hybridization in early gastrula animals (Figure 1). Initially, *Nvnotch* and *Nvdelta* expression is distributed in a “salt and pepper” pattern (Figure 1A,C), meaning that the cells that are expressing *Nvdelta* and *Nvnotch* are distributed throughout the ectoderm and appear like pepper granules mixed into a pile of salt. The *Nvnotch* “salt and pepper” pattern is slightly variable in that it appears patchy as if there are clusters of *Nvnotch* expressing cells distributed in the “salt and pepper” pattern (Figure 1C, yellow arrow). The expression of both genes expands over time, and both genes are ubiquitously expressed by the late gastrula stage (Figure 1B,D). Interestingly, within the ubiquitous *Nvdelta* expression, there is a “salt and pepper” distribution of cells that appear enriched for *Nvdelta* (Figure 2B, white arrows). Based on the spatiotemporal expression patterns previously reported [26] and extended here, *Nvnotch* and *Nvdelta* expression is consistent with the earliest onset of cellular differentiation.

### *Nvnotch* inhibits expression of the neurogenic transcription factor *NvashA*

To determine if Notch signaling in *Nematostella* functions to regulate cellular differentiation, we chose to characterize the effects of NvNotch activity on the expression of the previously identified neural differentiation gene *NvashA*. Previous reports showed that continuous treatment with DAPT for 72 hours resulted in an upregulation of *NvashA*[26]*,* but this study did not characterize earlier DAPT phenotypes. We assayed gastrula treated with 10 μM DAPT for *NvashA* expression by mRNA *in situ* hybridization and qPCR (Additional file 1; Figure 2)*. NvashA* expression levels increased by approximately two-fold in DAPT treated animals (Additional file 1; Figure 2G). Because DAPT does not directly inhibit Notch signaling, we were concerned that the DAPT *NvashA* phenotype may be caused by a disruption of a pathway other than Notch. To confirm that Notch signaling specifically inhibits *NvashA* expression, we generated splice-blocking MOs directed against the *Nvdelta* ligand and the *Nvnotch* receptor (Additional file 2A). The splice-blocking *Nvnotch* MO results in *Nvnotch* mRNAs containing stop codons that result in a premature truncation of the Notch intracellular domain (data not shown). Injection of the *Nvnotch* splice-blocking MO resulted in a cell that appeared to express relatively higher levels of *NvashA* compared to control (compare Figure 2A and D to B and E), a two-fold increase in *NvashA* expression measured by qPCR (Figure 2G), and a 60% increase in the number of *NvashA* positive cells (Figure 2H). The similar increase in *NvashA* expression observed in DAPT-treated and *Nvnotch* MO-injected animals suggest that the *NvashA* phenotype observed in DAPT-treated animals is specifically due to inhibition of NvNotch. A splice-blocking MO generated against *NvDelta* generates a miss-spliced transcript that encodes an *Nvdelta* transcript only missing the MNNL domain present in the extracellular region of the protein (Additional file 2A; data not shown). Injection of the *Nvdelta* splice-blocking MO results in an approximate 1.6-fold increase in *NvashA* expression (Figure 2G). This demonstrates that NvNotch and NvDelta are both required to repress *NvashA* in the embryonic ectoderm.

**Figure 3:**
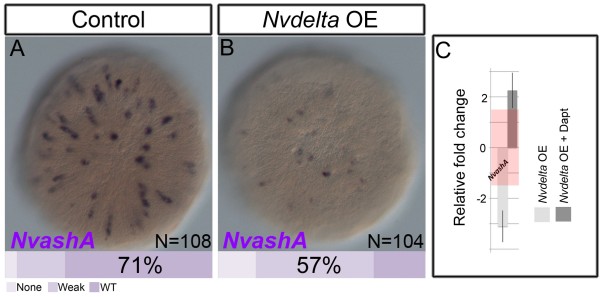
***Nvdelta *activates *Nvnotch *activity to suppress *NvashA*. (A-B)** Shown are Aboral views of *NvashA* expression in control **(A)** and *Nvdelta* overexpressing (OE) **(B)** animals. Phenotypic classes were scored as no expression, weak, wild-type (WT) levels, and strong expression. The key is shown in the image and bars at the base of each image represent the percentage of animals in each phenotypic class. **(C)** Relative fold change of *NvashA* and previously identified *Nvasha* neural gene targets and in animals overexpressing *Nvdelta* (light grey bars) and animals that are overexpressing *Nvdelta* and treated with DAPT (dark grey bars). Red rectangle denotes relative fold change −1.5 to 1.5, which corresponds to no change in relative expression.

To further confirm that Notch activity functions to repress *NvashA*, we used two approaches to overactivate Notch activity. First, we mimicked constitutively active Notch by injecting an mRNA encoding the *Nvnotch* intracellular domain fused in frame to the *venus* coding sequence (*Nvnicd:venus*) [37]. We observed NvNicd:Venus nuclear localization (Additional file 2D), and a nearly complete repression of *NvashA* expression as detected by mRNA *in situ* hybridization (Compare Figure 2A and D to C and F), and an approximate six-fold reduction in *NvashA* levels as detected by qPCR (Figure 2G). Second, we overactivated Notch activity by injecting mRNA encoding for the full length *Nvdelta* gene fused to the *venus* reporter (*Nvdelta:venus*). Overexpression of *Nvdelta* showed lower levels of *NvashA* expression by mRNA *in situ* hybridization (Figure 3). We observed weak *NvashA* expression in 57% of the *Nvdelta:venus* injected animals (Figure 3A,B) and an approximate three-fold reduction in *NvashA* expression as measured by qPCR (Figure 3C, light grey bar). To determine if the suppression of *NvashA* by *Nvdelta* required NvNotch, we treated *Nvdelta:venus* injected animals with DAPT. Treating *Nvdelta:venus* injected animals with DAPT resulted in a two-fold upregulation of *NvashA* (Figure 3C, dark grey bar). This is consistent with the previously observed phenotypes following DAPT treatment and *NvNotch* MO injection (Figure 2), and suggests that NvNotch acts to inhibit *NvashA* expression when activated by interactions with NvDelta*.*

### Notch activity suppresses neurogenesis through repression of *NvashA* expression

**Figure 4:**
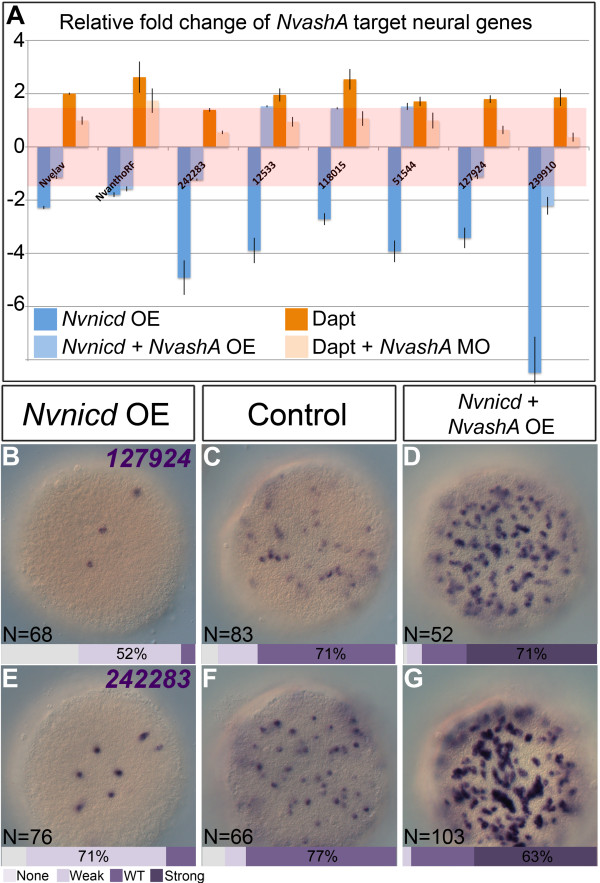
***Nvnotch *suppresses neurogenesis by regulating *NvashA *expression. (A)** Relative expression levels of *NvashA* target genes in animals with overexpressing (OE) *Nvnicd* (dark blue bars), overexpressing *Nvnicd* and *NvashA* (light blue bars), animals treated with DAPT (dark orange bars), and DAPT treated animals injected with the *NvashA* morpholino (MO) (light orange bars). Red rectangle represents relative fold change –1.5 to 1.5, which corresponds to no change in relative expression. Each treatment was repeated at least three times. **(B-G)** Aboral views of mRNA *in situ* images from two *NvashA* neural target genes are shown. Animals with overactive *Nvnotch***(B,E)**, control **(C,F)**, and both overactive *Nvnotch* and overactive *NvashA***(D,G)** are shown. Animals in (B-G) were quantified into phenotypic classes based on having no, weak, wild-type (WT)-like, or strong expression levels. The key is shown in the image and bars at the base of each image represent the percentage of animals in each phenotypic class.

To determine if changes in *NvashA* levels downstream of Notch activity correspond to changes in *NvashA*-dependent neurogenesis*,* we assayed for changes in expression of the previously identified *NvashA* neural target genes [30]. Overactivation of Notch activity by either injection of the full length *Nvdelta:venus* or injection of the *Nvnicd:venus* construct resulted in a dramatic downregulation of *NvashA* neural target genes (Figure 4A,D,G, dark blue bars; Additional file 3, light grey bars). Co-injection of *NvashA:venus* mRNA with the *Nvnicd:venus* mRNA was sufficient to suppress the reduction of neural gene expression phenotype resulting from overactivation of Notch activity (Figure 4C,F,G, light blue bars). Many of the *NvashA:venus Nvnicd:venus* co-injected embryos assayed by *in situ* hybridization showed neural gene expression phenotypes consistent with the increased number of neurons observed when *NvashA* is expressed alone (Figure 4C,F) [30]. Treatment with DAPT increased the levels of neural gene expression (Figure 4A, dark orange bars). Co-injection of the *NvashA* translation-blocking MO [30] suppresses the DAPT induced upregulation of neural gene expression (Figure 4A, light orange bars). These data suggest that Notch activity suppresses *NvashA*-dependent neurogenesis primarily through the specific inhibition of *NvashA* expression rather than broadly targeting downstream genes expressed in differentiating neurons.

### Post-embryonic treatment with DAPT increases *NvashA* expression in the larval ectoderm and endoderm

**Figure 5:**
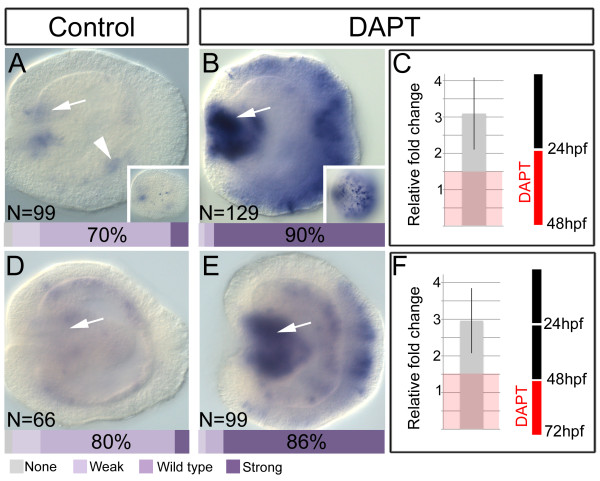
**DAPT treatment increases *NvashA *expression in the planula larva. (A-C)** Forty-eight hours post fertilization (hpf) animals either treated with control DMSO **(A)** or with DAPT **(B-C).** (A) *NvashA* expression in control animals is detected in the developing pharynx (arrow), in the endoderm (arrowhead), and in the ectoderm (inset). (B) Treatment with DAPT increases *NvashA* expression in each tissue. (C) Quantitative (q)PCR analysis reveals a three-fold increase in the relative levels of *NvashA* in DAPT-treated animals. **(D-F)** Seventy-two hpf animals either treated with control DMSO (D) or with DAPT (E-F). (D) *NvashA* expression in control animals is detected in the developing pharynx (arrow) and in the endoderm. (E) Treatment with DAPT increases *NvashA* expression in each tissue. (F) qPCR analysis reveals a three-fold increase in the relative levels of *NvashA* in DAPT-treated animals. The key in (C) and (F) shows that animals were grown in normal 1/3X sea water (black line between time intervals) or in the presence of DAPT (red line between time intervals). Animals in (A,B,D,E) were quantified into phenotypic classes based on having no, weak, wild-type-like, or strong expression levels. The key is shown in the image and bars at the base of each image represent the percentage of animals in each phenotypic class. Red box in (C and F) indicates the region between 0 and 1.5-fold change, which we consider to indicate no change in expression. All animals are shown in a lateral view with the oral side to the left.

We wanted to test whether Notch activity regulates *NvashA* at later developmental stages independently of the earlier roles described above. In order to disrupt Notch signaling at later stages without disrupting Notch signaling at early stages we opted to use DAPT treatments. Although DAPT treatment may not specifically disrupt Notch signaling, the increase in *NvashA* following treatment with DAPT or injection of the *Nvnotch* MO in the embryo are identical (Figure 1), which suggests the DAPT *NvashA* phenotype is due to a disruption of Notch signaling. We performed two DAPT treatments (Figure 5). The first treatment began at the late gastrula stage and continued for 24 hours into the early planula larval stages (Figure 5A-C). We detected *NvashA* expression in the forming pharynx (Figure 5A, arrow), in a “salt and pepper” pattern in the ectoderm (Figure 5A, inset), and some weak staining in a “salt and pepper” pattern within the endoderm in control planulae (Figure 5A, arrow head). Treatment with DAPT resulted in an increase in pharyngeal staining (Figure 5B, arrow) and an increase in the number of ectodermal cells expressing *NvashA* (Figure 5B, inset). It was difficult to be certain that endodermal *NvashA* was increased because of the strong ectodermal expression, but it appears as if there is an expansion of *NvashA* expression in the endoderm as well. We were also able to classify animals into groups of animals having no, weak, normal wild-type, or strong *NvashA* expression for both control and DAPT-treated animals. In control animals, approximately 70% of the animals had wild-type levels of *NvashA* expression, and only approximately 10% of the animals had strong expression of *NvashA.* In DAPT-treated animals 90% of the animals displayed the strong expression phenotype. We also observed a three-fold increase in *NvashA* expression in DAPT-treated animals by qPCR (Figure 5C). We also treated animals with DAPT from 48 to 72 hpf, which ensured animals were all within the larval stages of development during the treatment (Figure 5D-F). *NvashA* expression in control 72 hpf planulae was detected in the pharynx and forming mesentery structures (Figure 5D, arrow) and in a “salt and pepper” endodermal pattern. We did not detect any ectodermal *NvashA* expression in 72 hpf animals. Animals treated with DAPT from 48 to 72 hpf showed a strong increase in *NvashA* in the forming pharynx and mesenteries (Figure 5E, arrow), and the endoderm has an increase in *NvashA* expression levels. As before, we could easily group phenotypic classes for *NvashA* expression: in control animals, 80% of the animals showed wild-type expression levels and only approximately 7% showed the strong *NvashA* expression phenotype (Figure 5D). However, in the DAPT-treated animals 86% of the animals displayed the strong *NvashA* expression phenotype (Figure 5E). DAPT-treated animals also had an approximate three-fold increase in *NvashA* expression levels by qPCR (Figure 5F). These data demonstrate that DAPT treatment promotes an increase in *NvashA* at later stages, and that similar mechanisms regulate both embryonic and larval differentiation. Moreover, these results argue that the dynamic expression patterns observed for *Nvnotch* and *Nvdelta* (ectoderm in early embryo and moving into the endoderm in larval stages [26]) supports the hypothesis that *Nvnotch* regulates cellular differentiation in multiple tissues throughout development in *Nematostella.*

### *Nvnotch* broadly inhibits expression of genes associated with neuronal differentiation

**Figure 6:**
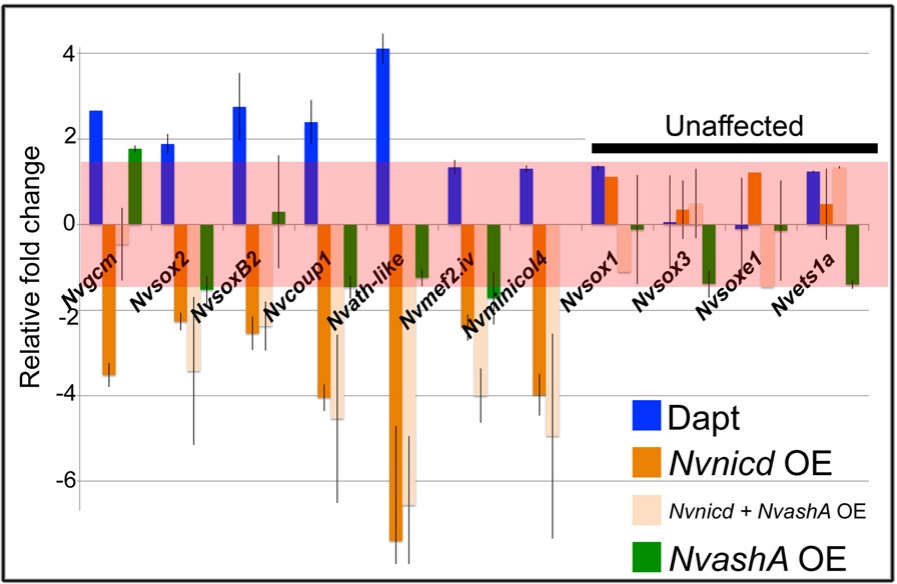
***Nvnotch *regulates “salt and pepper” differentiation genes.** Relative fold change of “salt and pepper” genes in animals following treatment with DAPT (blue bars), injection with *Nvnicd* (dark orange bars), injection with *Nvnicd* and *NvashA* (light orange bars), or *NvashA* alone (green bars). Red rectangle denotes relative fold change −1.5 to 1.5, which indicates no change in relative expression. “salt and pepper” differentiation genes are suppressed by *Nvnotch* activity while genes with broad expression domains are unaffected by any of our treatments. OE, overexpressing.

Lastly, we wondered if Notch activity might influence expression levels of other differentiation genes unrelated to *NvashA*. We used previously described differentiation genes, *Nvgcm, Nvsoxb2, Nvsox2, Nvmef2.iv,* and *Nvminicol4*[31,38-40], as well as two recently identified genes, *Nvcoup1* and *Nvath-like1* (Figure 6)*,* that, like *NvashA*, are all expressed in a “salt and pepper” pattern. It should be noted that all of these genes are associated with neuronal differentiation, though only *Nvmef2.iv* and *Nvminicol4* have been definitively linked to neural development. They regulate formation of the cnidocyte neural cell type [39,40]. As we observed for *NvashA,* inhibiting Notch activity by treating with DAPT (Figure 6, blue bars) or injecting the *Nvnotch* MO (Additional file 4, green bars) increased expression levels for nearly all the “salt and pepper” genes assayed. The only genes assayed that showed no significant increase in expression levels following treatment with DAPT were *Nvmef2.iv* and *Nvminicol4,* though *Nvminicol4* was upregulated following *Nvnotch* MO injection (Additional file 4). We also included *Nvsox1*, *Nvsox3, Nvsoxe1*, and *Nvets1a* because they are expressed in distinct broad domains rather than in a “salt and pepper” pattern, which suggests that they are involved in patterning regional domains rather than differentiation. Expression levels of the “broadly expressed” genes did not change following DAPT treatment or injection of the *Nvnotch* MO. Overactivation of Notch signaling by injecting *Nvnicd:venus* suppressed expression of all of the “salt and pepper” genes (Figure 6, dark orange bars), including *Nvmef2.iv* and *Nvminicol4*. Again, the broadly expressed genes were unaffected by *Nvnicd* injection.

To confirm these genes are independent of *NvashA*, we attempted to rescue the loss of “salt and pepper” gene expression resulting from overactivation of Notch signaling by co-injecting the *Nvnicd:venus* and the *NvashA:venus* constructs (Figure 6, light orange bars). Only *Nvgcm* was rescued by expression of *NvashA.* This suggests that, with the exception of *Nvgcm,* the “salt and pepper” genes are not targets of *NvashA.* Therefore, we suggest Notch activity broadly regulates expression of genes associated with neural differentiation in the *Nematostella* embryo.

### The non-canonical Notch signaling pathway inhibits *NvashA* expression

**Figure 7:**
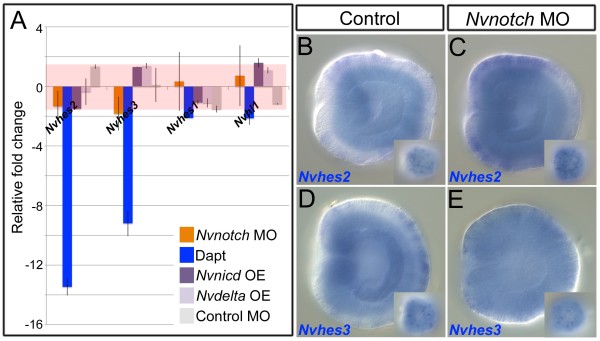
***Nvnotch *does not regulate *Nvhes *expression in the *Nematostella *embryo. (A)** Average relative fold change of *Nvhes* gene expression in animals injected with *Nvnotch* morpholino (MO; orange bars), treated with DAPT (blue bars), injected with *Nvnicd:venus* (dark purple bars), injected with *Nvdelta:venus* (light purple bars), or a control MO (grey bars. Red rectangle covers the region where the relative fold change ratio is equal to −1.5 to 1.5 and corresponds to no change in relative expression level. **(B-E)** Lateral views of late stage gastrula expressing *Nvhes2***(B-C)** or *Nvhes3***(D-E)**. Oral is to the left. Deep focal plane is shown and superficial focal plane is shown in inset. We observed no discernable difference in *Nvhes2* or *Nvhes3* expression by *in situ* analysis between wild-type and *Nvnotch* MO injected animals. We scored N >80 embryos for each treatment. OE, overexpressing.

Suppression of *NvashA* by activated Notch signaling can occur through the canonical (*suH* and *hes* gene-dependent), the non-canonical (*suH* and *hes* gene-independent), or through both pathways. We tested the putative contributions of the canonical and non-canonical pathways in *Nematostella*. First, we tested if *Nvnotch* regulated the expression of *Nvhes* genes. Four *Nvhes* genes, *Nvhes1, 2, 3, Nvhl1*, are expressed in *Nematostella* embryos and could potentially be regulating *NvashA*[26]. However, only *Nvhes2* and *Nvhes3* expression is detected by mRNA *in situ* hybridization in the early gastrula when the earliest onset of differentiation of *NvashA* positive cells is occurring [26]. We compared changes in expression for each of these genes using qPCR following treatment with DAPT (Figure 7, blue bars), injection of the *Nvnotch* MO (Figure 7, orange bars), and following injection of the *Nvnicd:venus* mRNA (Figure 7, purple bars). Treatment with DAPT resulted in an approximate two-fold reduction in *Nvhes1* and *Nvhl1* levels. The *Nvhes2* and *Nvhes3* genes both showed a greater than eight-fold reduction in expression following DAPT treatment (Figure 7A, blue bars). However, *Nvnotch* MO injected animals showed no change in *Nvhes1* or *Nvhl1* expression, and a relatively minor decrease in *Nvhes2* and *Nvhes3* levels (Figure 7A, orange bars). We failed to detect any reduction of *Nvhes2* or *Nvhes3* in *Nvnotch* morphants by mRNA *in situ* hybridization (Figure 7B-E). Because the *NvashA* phenotype resulting from injection of the *Nvnotch* MO is as severe as treatment with DAPT (Figure 1), and there is little wild-type *Nvnotch* transcript in the *Nvnotch* morphant animals (Additional file 2A), we believe the Notch MO to be highly efficient. However, we were concerned that low levels of *Nvnotch* activity may be sufficient to promote *Nvhes* gene expression in the embryo. To address this we overactivated Notch signaling by injecting the *Nvnicd:venus* and *Nvdelta:venus* constructs, which should increase *Nvhes* expression if the canonical pathway was intact. We observed no significant change for *Nvhes1-3* and only a minor increase in *Nvhl1* expression following injection of *Nvnicd:venus* (Figure 7E, purple bars)*.* Similarly, injection of the *Nvdelta:venus* mRNA failed to induce expression of any of the *Nvhes* genes. Thus, our data suggest that, although DAPT treatment reduces the expression levels of *Nvhes1-3* or *Nvhl1* in *Nematostella* embryos, the observed downregulation is *Nvnotch*-independent.

**Figure 8:**
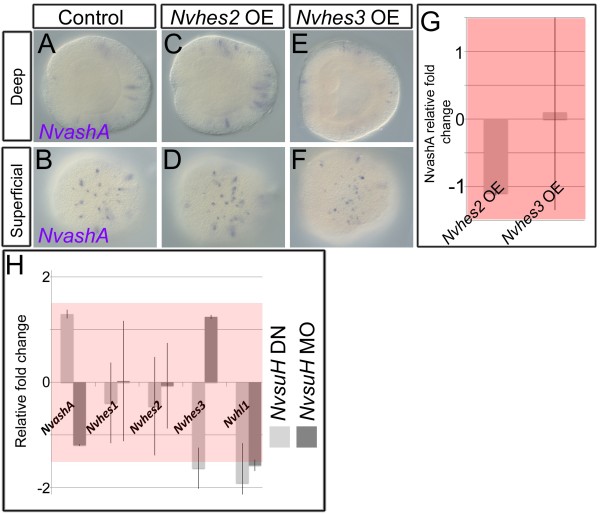
***Nvhes2 *and *Nvhes3 *overexpression does not repress *NvashA *expression. (A-F)** Lateral views of embryos expressing *NvashA*; oral is to the left. There is no discernable difference in *NvashA* expression in control (A-B), *Nvhes2* overexpressing (OE) **(C-D)**, or *Nvhes3* (E-F). N >65 scored for each experiment. **(G)** Relative fold change of *NvashA* in embryos treated overexpressing *Nvhes2* or *Nvhes3*. **(H)** Average relative fold change of *NvashA*, neural genes, and *Nvhes* genes in animals injected with the *NvsuH* morpholino (MO; dark grey bars) or a dominant negative *NvsuH* (DN; light grey bars). Red rectangle denotes relative fold change −1.5 to 1.5, which indicates no change in relative expression. Each injection was repeated three times.

Even though *Nvnotch* does not regulate *Nvhes* genes we still wanted to test if *NvsuH* regulated *Nvhes* genes, and if *Nvhes* genes were sufficient to suppress *NvashA* expression. *Nvhes2* and *Nvhes3* are the only two *Nvhes* genes that have expression that initiates in the early embryo when the first cellular differentiation is observed in *Nematostella*. We overexpressed *Nvhes2* and *Nvhes3* by injecting *venus:Nvhes2* and *venus:Nvhes3* mRNAs. *Nvhes2* or *Nvhes3* overexpression did not result in any changes in the levels of *NvashA* as detected by mRNA *in situ* hybridization (Figure 8A-F) or qPCR (Figure 8G). We tested if *NvsuH* regulated *Nvhes* genes or *NvashA* by injecting both an *NvsuH* MO and a dominant negative *NvsuH*[26]. Neither of these manipulations resulted in detectable changes of *Nvhes* or *NvashA* expression by qPCR (Figure 8H). These data suggest that the canonical Notch pathway does not regulate *NvashA*-dependent neural development in the early *Nematostella* embryo.

To determine if canonical Notch signaling could regulate the *NvashA*-independent “salt and pepper” expressed genes associated with cellular differentiation, we tested whether overexpressing *Nvhes2* or *Nvhes3* via injection of the *venus:Nvhes2* or *venus:Nvhes3* mRNA could suppress expression of the “salt and pepper” genes. We saw no change in the expression levels by qPCR for any of the “salt and pepper” genes assayed here (Additional file 4, light and dark blue bars). Thus, it appears that non-canonical Notch signaling broadly suppresses expression of genes that promote neural differentiation in *Nematostella* embryos.

## Discussion

### Model of Notch signaling in *Nematostella*

Our data show that NvNotch is activated by NvDelta to regulate cellular differentiation in *Nematostella*, but based on our observations here it is likely that Notch activity in *Nematostella* regulates the competence of cells to respond to a variety of instructive differentiation cues. Elevated levels of Notch activity suppress differentiated cell markers, while decreased levels of Notch activity increase expression of differentiated cell markers (Figures 1 and 8; Additional file 1)*.* However, inhibition of Notch signaling is not sufficient to induce a total transformation of cells into differentiated cells. This suggests that Notch either acts at defined time points in the differentiation process or that Notch-independent instructive cues act to induce particular differentiated cell types. Our model predicts that the relative level of Notch activity and the amount of inductive signal coordinate to determine if differentiation will occur. Consistent with this prediction, extending treatment with DAPT to 3 days results in animals that have a more pronounced expansion of differentiated cell markers than our early shorter treatment [26]. One interpretation is that extended inhibition of Notch activity provides more opportunity for undifferentiated cells to encounter and respond to external inductive cues. Additionally, quantification of the number of cells expressing any one “salt and pepper” gene is not often reproducible from animal to animal (Figure 1H; unpublished observations) [30], suggesting that the mechanism governing the number of cells of a distinct cell type is somewhat stochastic. Taken together these observations argue that, in any given animal at a given time, there are variable numbers of cells competent to respond to distinct differentiation cues. Our data supports the hypothesis that the competence is in part regulated by *Nvnotch* activity.

Notch appears to function broadly to inhibit neural differentiation. We tested a number of genes that have been previously reported to be associated with differentiation during *Nematostella* development (Figure 6; Additional file 3). We found that inhibiting *Nvnotch* by injecting the *Nvnotch* MO or by treating with DAPT resulted in upregulation of the differentiated markers. Conversely, overactivation of Notch by overexpressing the *Nvnicd:venus* mRNA suppressed expression of the differentiation markers. The markers that we used (*NvashA, Nvsox2, Nvgcm, Nvsoxb2, Nvath-like, Nvmef2.iv, Nvminicol4, Nvcoup1-like*) are all predicted to be associated with neurogenesis and/or cnidocyte development in *Nematostella*, although (with the exception of *NvashA*, *Nvmef2.iv,* and *Nvminicol4*) none of them are confirmed regulators of neural development. Thus, we cannot conclude at this point if Notch broadly regulates expression of all differentiated cell types or specifically regulates neural development in *Nematostella*. Even if the differentiation genes we chose are specific to neural development we argue that they are independent of *NvashA*-dependent neural development. We show that, other than *Nvgcm*, none of the differentiation genes assayed here can be rescued when *NvashA* is overexpressed in animals with increased Notch activity (Figure 5). Also, we have not observed any co-expression of *Nvsoxb2* or *Nvsox2* with *NvashA* neural targets, and both *NvsoxB2* and *Nvsox2* are expressed in what appears to be many more cells than *NvashA*[38] (unpublished observation)*.* Thus, we are confident that Notch activity broadly inhibits expression of genes associated with neural differentiation, but cannot determine what other cell types might be regulated by Notch activity.

We also propose that Notch regulation of differentiation is a reiterative process during *Nematostella* development. Differentiation begins during the early gastrula stage of *Nematostella* development, but continues throughout embryonic and larval stages. The expression patterns of *NvashA* and other known developmental genes are known to be dynamic throughout these stages [30,31,36,38]. Expression of Notch signaling components appears to be enriched in tissues likely to be undergoing cellular differentiation during development. For example, the embryonic expression of *Nvnotch* and *Nvdelta* initiate in the ectoderm, and are maintained there until late planula stages (Figure 2) [26]. In early planula stages the endoderm begins to show expression of differentiated cell markers [31,36]. Endodermal expression of *Nvnotch* and *Nvdelta* are coincident with endodermal differentiation. *Nvnotch* and *Nvdelta* are expressed in the forming and growing tentacle buds [26] (unpublished observation), and expression in juvenile and adult polyps is maintained in the endodermal portion of the eight mesenteries [26], where constant differentiation of nematosomes is known to occur [41]. We also found that treating with DAPT for distinct time windows throughout larval development resulted in the increased *NvashA* expression (Figure 5). This suggests that the same or a similar mechanism controls *NvashA* expression at later time points and in different tissues (endoderm versus ectoderm) than during embryonic development. We would like to extend this temporal analysis to gene-specific knockdowns of *Nvnotch*. However, we focused this initial study on the early embryonic roles of *Nvnotch* because conditional knockdown of *Nvnotch* function specifically at later time points is still difficult in *Nematostella*. As the technology of conditional alleles to disrupt gene function specifically at distinct life stages in *Nematostella* advances, and as identification of genes that serve as markers for cells differentiated within distinct temporal windows are found, our model can be tested further. We predict that disrupting Notch activity in distinct temporal windows should disrupt only the cell types that are normally born within that time frame.

### Notch signaling pathway may have emerged to regulate metazoan cellular differentiation

The emergence of multicellular animals with specialized cell types had to require a mechanism to regulate whether cells differentiate or remain pluripotent. Notch has been shown to have a highly conserved role as a regulator of differentiation in nearly all bilaterian tissues. However, prior to this study it was unclear how Notch functioned in non-bilaterian animals, and thus there was little inference about ancestral Notch function. We show that non-canonical Notch signaling in the cnidarian sea anemone, *Nematostella vectensis*, broadly inhibits cellular differentiation during development. This provides a clear example of Notch regulating differentiation outside of Bilateria. Given how highly conserved the role for Notch as a regulator of differentiation appears, and the fact that core Notch components evolved specifically in metazoans, it is likely that Notch regulates differentiation in all metazoans. To fully support this hypothesis we need to reconstruct the function of Notch signaling in the common ancestor of all metazoans by characterizing the role of Notch in animals representing the earliest diverged metazoan lineage. The sister lineage to the rest of animals is still being debated, but the current consensus is that it is either Ctenophora or Porifera. Disruption of gene function in either of these groups has proven difficult, but we can infer putative function based on expression patterns. Expression of *notch* and *delta* homologs in the poriferan *A. queenslandica* initiates expression in a spatiotemporal pattern consistent with regulators of cellular differentation [25]. The *amqdelta* homologs appear to be expressed in differentiating and differentiated cell types consistent with the idea that they activate Notch to suppress differentiation in the surrounding cells, while having low Notch activity themselves [25]. The expression patterns of Notch signaling homologs in ctenophores are not known, and definitive homologs for *delta* have not been found. Thus, we cannot predict putative functions for Notch signaling in that lineage.

### Evolution of canonical Notch signaling

Our results suggest that canonical Notch signaling is not present in the cnidarian lineage and that the canonical pathway evolved in the stem of the bilaterian lineage. In *Nematostella*, gene-specific knockdown of *Nvnotch, NvsuH,* or overactivation of *Nvnicd* did not significantly affect expression levels of *Nvhes* genes, which are an important target of the canonical Notch signaling pathway. Overactivation of Notch signaling by overexpressing either *Nvnicd* or *Nvdelta* was sufficient to suppress expression of differentiated cell markers, but both failed to upregulate any of the *Nvhes* genes monitored (Figure 5). Furthermore, overexpression of *Nvhes2* or *Nvhes3* failed to suppress *NvashA* or other genes associated with cellular differentiation (Figure 5; Additional file 4). In addition, the expression of *Nvhes* homologs throughout *Nematostella* development are inconsistent with the notion that they are targets of *Nvnotch* signaling. Most *Nvhes* genes show minimal overlap with *Nvnotch* expression outside of the embryonic ectoderm [26]. Three exceptions to this are *Nvhes3 and Nvhl1,* which overlap with *Nvnotch* expression in the oral ectoderm and aboral ectoderm during planula stages [26], and *Nvhes1*, which overlaps with the *Nvnotch* expression in the planula endoderm. However, *Nvhes1* expression appears ubiquitous in the planula stages, whereas *Nvnotch* expression becomes limited to the endoderm, suggesting that the *Nvhes1* expression is regulated by factors other than *Nvnotch*. The reported expression of *NvsuH* is also inconsistent with the idea that canonical Notch signaling regulates differentiation. *NvsuH* is not expressed in the differentiating ectoderm at the onset of cellular differentiation in the early gastrula when expression of *NvashA* and the “salt and pepper” genes is initiated [26]. However, *NvsuH* is expressed ubiquitously later in the planula larval stages.

A closer examination of the phylogenetic distribution of canonical Notch signaling components in the three published cnidarian genomes also supports the lack of an intact canonical Notch pathway in cnidarians [4,23,24]*.* Previous analysis suggested that the cnidarian-bilaterian common ancestor was the first animal with a compliment of genes that participate in canonical Notch signaling [19]. However, the cnidarian homologs of the transcriptional co-activator *mastermind* that is recruited to activate *hes* expression are only weakly conserved at best with bilaterian homologs [1,19]. Moreover, SuH also interacts with the SMRT co-repressor to suppress expression of *hes* homologs when Notch signaling is not active. *smrt* homologs have not been identified in any of the currently published cnidarian genomes [4,19,23,24].

It should be noted that most of the *Nvhes* genes are severely downregulated following DAPT treatment (Figure 5) [26]. However, our data argue that the DAPT-induced *Nvhes* phenotypes occur independently of *Nvnotch*. The current draft of the *Nematostella* genome describes only a single *Nvnotch* gene. However, there are additional single pass transmembrane proteins that, like *Nvnotch*, have EFG repeats in their extracellular domain (unpublished observation) [24]. The intracellular domains of these proteins lack the typical intracellular domains linking Notch signaling to *hes* gene regulation [19,26], but because the γ-secretase complex is believed to cleave most single pass transmembrane signaling proteins, it is reasonable to hypothesize that DAPT is affecting one or more of these “Notch-like” proteins, and that they may regulate *hes* expression. Given that activation of *hes* expression is a hallmark of canonical Notch signaling, we speculate that some aspect of *hes* biology underlies the emergence of the canonical pathway. One explanation could be based on the fact that *hes* genes function as oscillators that promote cell proliferation [13,42]. Interestingly, we observe *Nvhes2* expression in proliferating cells (unpublished observation). Because high Notch activity often suppresses differentiation, perhaps incorporating regulation of proliferation downstream of Notch activity provided a mechanism to both suppress differentiation and promote proliferation. This is consistent with the observation that canonical Notch activity in the development of many bilaterian tissues is often associated with maintaining tissue-specific stem cells [8].

To verify that canonical Notch signaling is not intact in the cnidarian-bilaterian ancestor gene specific functional studies need to be conducted in other cnidarian species. Additional analyses need to be done in *Nematostella* once tools emerge to investigate roles for Notch signaling specifically during post-embryonic development. Currently, attempting to interpret late-stage phenotypes in morphant animals is complicated because it is unclear how early disruption of *Nvnotch* influences later development. Temporal-specific treatments with DAPT would not be informative because we showed that the responses of *Nvhes* genes to DAPT in the embryo are *Nvnotch*-independent phenotypes.

## Conclusions

Based on our functional analysis in the cnidarian *Nematostella vectensis* and previous pharmacological experiments in other cnidarian species, we propose that the Notch signaling pathway regulated cellular differentiation in the cnidarian-bilaterian ancestor. This argues that the role of Notch as a regulator of cellular differentiation evolved prior to the last common ancestor of bilaterian animals. Functional studies are required in other non-bilaterian lineages to reconstruct the role of Notch signaling at more basal nodes in the metazoan phylogeny. Because all components of canonical Notch signaling likely did not evolve until the cnidarian-bilaterian common ancestor, a full complement of canonical signaling components only exists in the bilaterians, and because canonical Notch signaling is not required for *Nvnotch* to regulate embryonic neural differentiation in *Nematostella*, we speculate that non-canonical Notch signaling is the ancestral notch mechanism and that the canonical pathway likely evolved specifically in the bilaterian lineage.

## Abbreviations

hpf: Hours post-fertilization; MO: morpholino; (q)PCR: (quantitative) polymerase chain reaction.

## Competing interests

The authors declare that they have no competing interests.

## Authors’ contributions

MJL conceived and carried out the generation of constructs, collection of data, and data analysis. MJL and MQM carried out animal injections. MJL and MQM wrote the manuscript. Both authors read and approved the final manuscript.

## Supplementary Material

Additional file 1 **DAPT treatment upregulates *NvashA.*** (A-D) Shown are lateral views of embryos expressing *NvashA*. Oral is to the left. DAPT-treated animals have higher levels of *NvashA* expression. Phenotypic classes we scored as being wild-type, strong, weak, or no *NvashA* expression. Key is shown in image and bars at the base of each image represent the percentage of animals in each phenotypic class. Click here for file

Additional file 2 **Control experiments.** (A) Splice blocking efficiency for each splice MO used in this study is shown. (B-D) Injection of mRNAs encoding for the *Nvnicd:venus* (B), *venus:Nvhes2* (C), and *venus:Nvhes3* (D) resulted in translated protein and can be detected in the nuclei of the developing embryo. Click here for file

Additional file 3 **Relative fold change of *NvashA *neuronal targets in *Nvdelta *OE animals.** Relative fold change of *NvashA* neural target genes in animals overexpressing the *Nvdelta:venus* mRNA (light grey bars) or overexpressing the *Nvdelta:venus* mRNA and treated with DAPT (dark grey bars). Red box indicates region where fold change ratio is between −1.5 and 1.5 indicating no change in expression. Click here for file

Additional file 4 **Relative fold change of “salt and pepper” genes in *Nvnotch *morphant and *Nvhes *overexpressing animals.** Relative fold change of “salt and pepper” and broad domain expressed controls are shown for animals injected with the *Nvnotch* MO (green bars), *venus:Nvhes2* (light blue bars), or *venus:Nvhes3* (dark blue bars). Each injection was repeated at least three times. Red box indicates region where fold change ratio is between −1.5 and 1.5 indicating no change in expression. Click here for file
