# Supplementary figures and images for: Non-canonical Notch signaling represents an ancestral mechanism to regulate neural differentiation
Source: EvoDevo. 2014 Sep 19;5:30. doi: 10.1186/2041-9139-5-30 (PMC4335385; doi:10.1186/2041-9139-5-30)

# Control

# DAPT

## Deep

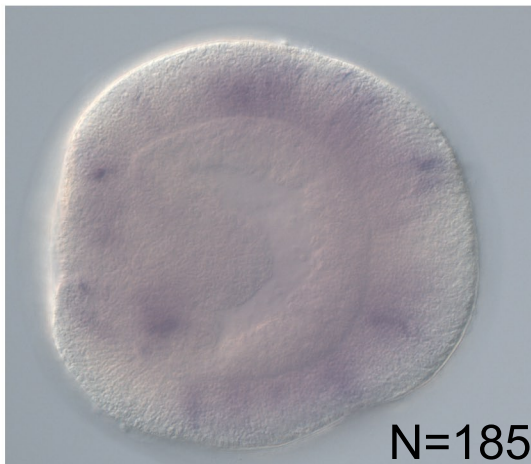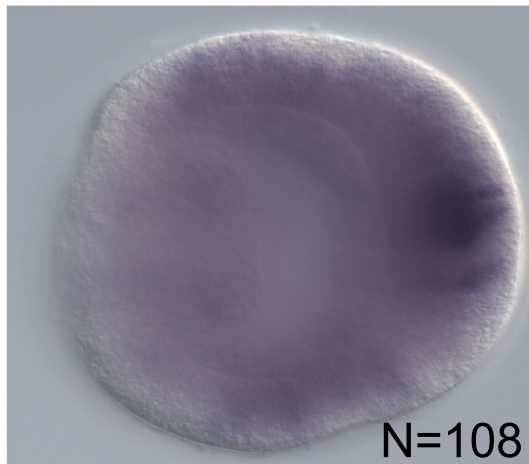

## Superficial

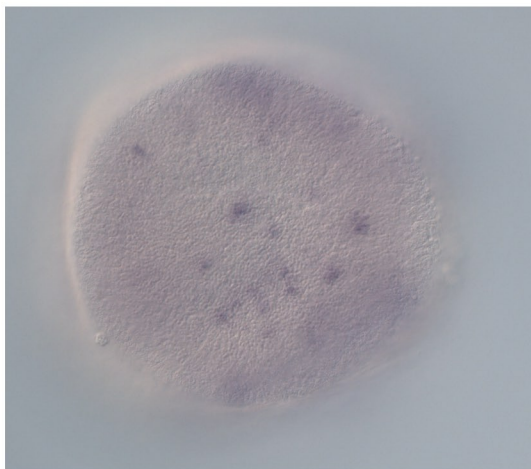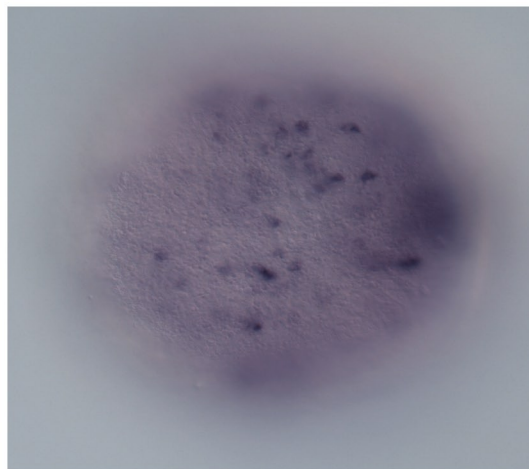

None Weak WT Strong

Supplement: Additional file 1 — DAPT treatment upregulates NvashA. (A-D) Shown are lateral views of embryos expressing NvashA. Oral is to the left. DAPT-treated animals have higher levels of NvashA expression. Phenotypic classes we scored as being wild-type, strong, weak, or no NvashA expression. Key is shown in image and bars at the base of each image represent the percentage of animals in each phenotypic class. [file 2041-9139-5-30-S1.pdf]

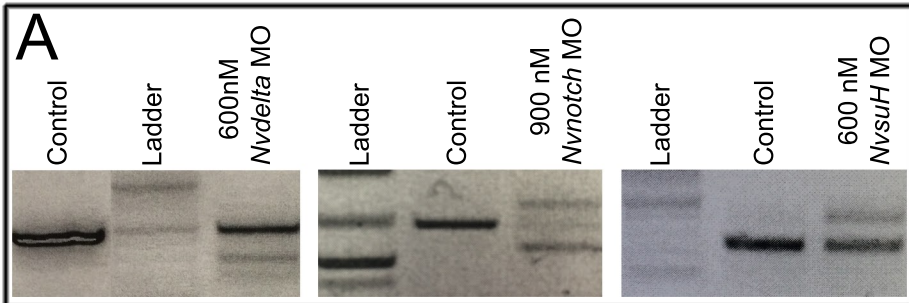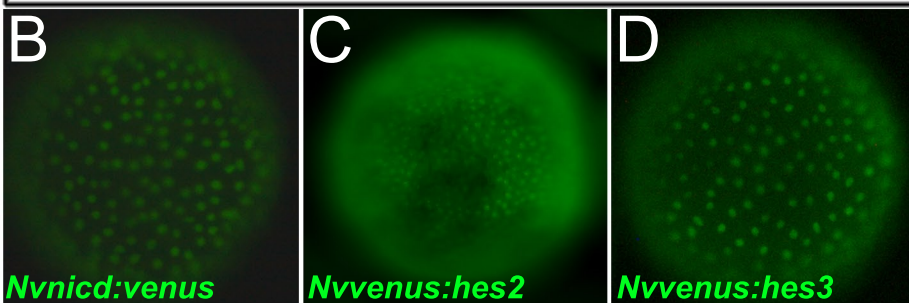

Supplement: Additional file 2 — Control experiments. (A) Splice blocking efficiency for each splice MO used in this study is shown. (B-D) Injection of mRNAs encoding for the Nvnicd:venus (B), venus:Nvhes2 (C), and venus:Nvhes3 (D) resulted in translated protein and can be detected in the nuclei of the developing embryo. [file 2041-9139-5-30-S2.pdf]

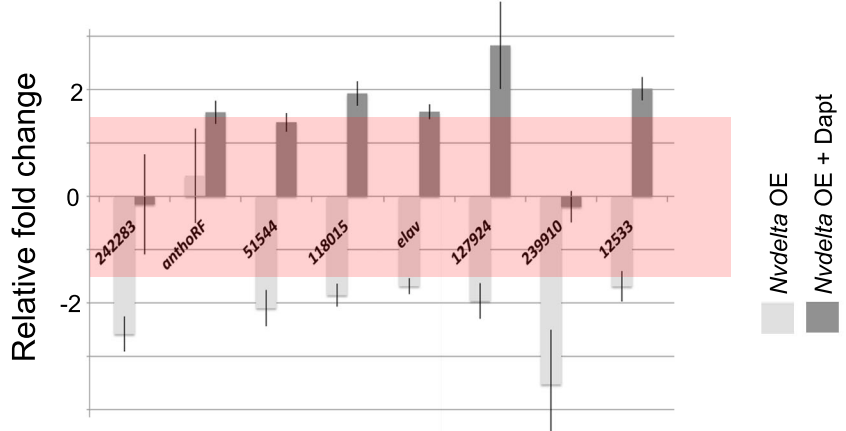

Sup Fig 3

Supplement: Additional file 3 — Relative fold change of NvashA neuronal targets in Nvdelta OE animals. Relative fold change of NvashA neural target genes in animals overexpressing the Nvdelta:venus mRNA (light grey bars) or overexpressing the Nvdelta:venus mRNA and treated with DAPT (dark grey bars). Red box indicates region where fold change ratio is between −1.5 and 1.5 indicating no change in expression. [file 2041-9139-5-30-S3.pdf]

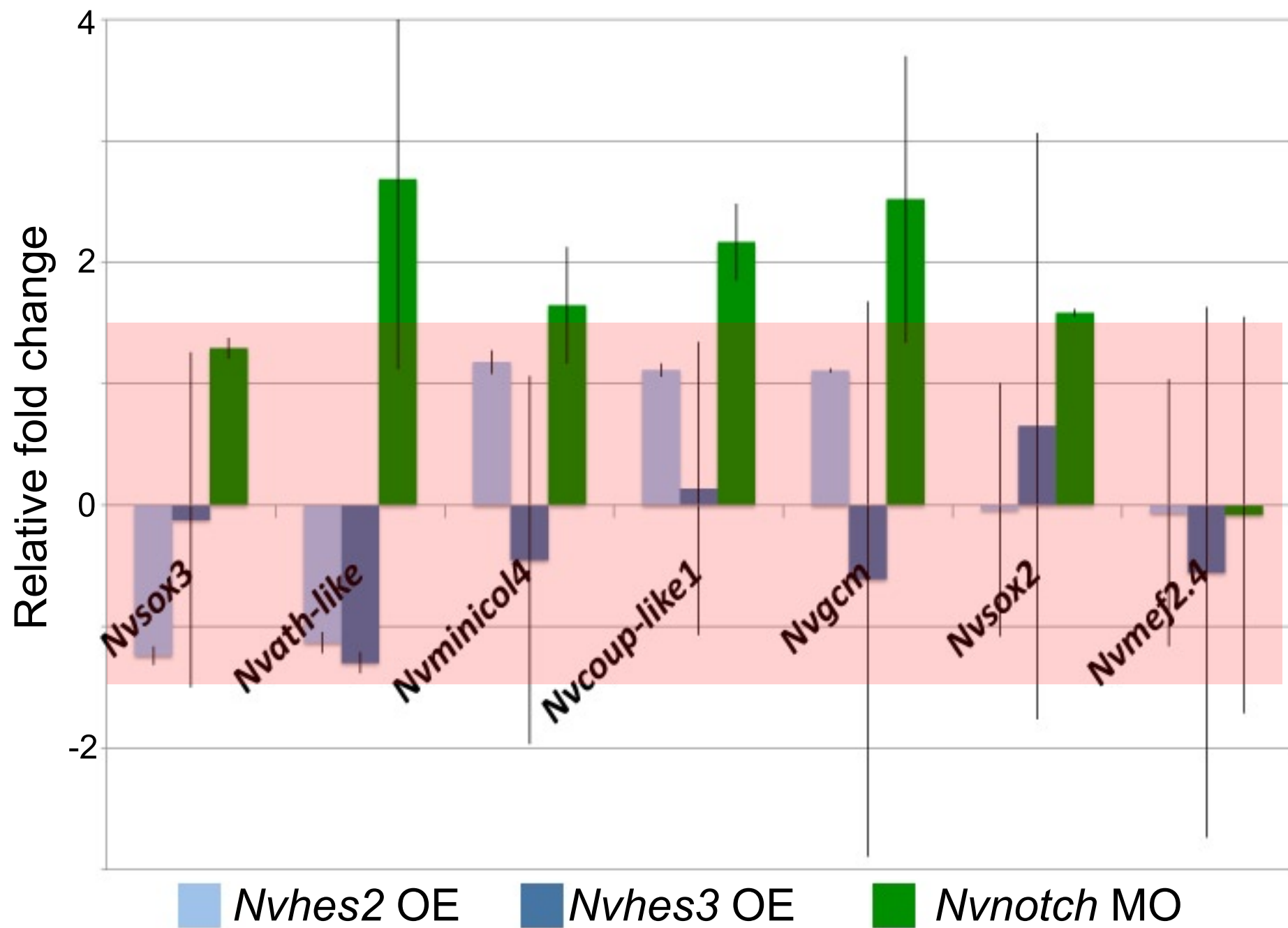

Supplement: Additional file 4 — Relative fold change of “salt and pepper” genes in Nvnotch morphant and Nvhes overexpressing animals. Relative fold change of “salt and pepper” and broad domain expressed controls are shown for animals injected with the Nvnotch MO (green bars), venus:Nvhes2 (light blue bars), or venus:Nvhes3 (dark blue bars). Each injection was repeated at least three times. Red box indicates region where fold change ratio is between −1.5 and 1.5 indicating no change in expression. [file 2041-9139-5-30-S4.pdf]
